# Causative Pathogens in Surgical Drain Infections and Antibiotic Resistance Profiles of These Pathogens: Growing Frequency of Resistance Among the Enterobacteriaceae Family

**DOI:** 10.7759/cureus.36431

**Published:** 2023-03-20

**Authors:** Ese Basbulut, Gultekin Ozan Kucuk

**Affiliations:** 1 Department of Microbiology and Clinical Microbiology Laboratory, Samsun Training and Research Hospital, Samsun, TUR; 2 Department of General Surgery, University of Health Sciences, Samsun Training and Research Hospital, Samsun, TUR

**Keywords:** surgical site infection, staphylococcus, enterobacteriaceae, drain culture, antibiotic resistance

## Abstract

Introduction

Surgical drain infections (SDIs) and antibiotic resistance profiles of these infectious pathogens are the issues that need to be emphasized. This study aimed to identify microorganisms isolated from drain cultures and determine antibiotic resistance rates among these microorganisms.

Materials and methods

The drain culture results of patients analyzed between January 2008 and January 2020 were included in the study. Data such as microorganisms grown in drain cultures, antibiotic resistance rates, and demographic information of patients were evaluated.

Results

Three hundred forty-six isolates were analyzed from the drain cultures of 279 patients. The mean age of the patients was 62.82 ± 17.77 years. Polymicrobial growth was detected in samples from 49 (18%) patients. The most frequently isolated microorganisms were pathogens belonging to the Enterobacteriaceae family (44%) and to Staphylococcus species (spp.) (20%). As shown by our results, the frequency of *Staphylococcus spp*. decreased in the last four years, whereas the frequency of *Enterococcus* increased. In terms of drug resistance, the highest rate of resistance among the isolates was to ampicillin (Enterobacteriaceae family), followed by gentamicin (*Acinetobacter species.*), cefepime (*Pseudomonas spp*.), penicillin (*Staphylococcus spp*.), and ciprofloxacin (*Enterococcus spp*.). In the Enterobacteriaceae family, 49% of the isolates were resistant to extended-spectrum beta-lactamases, and 17% were resistant to carbapenems. Methicillin resistance was detected in 55% of *Staphylococcus aureus*, and vancomycin resistance was found in 11% of Enterococcus.

Conclusions

In drain cultures for SDIs, information on the causative pathogens, in addition to the antibiotic resistance rates of these pathogens, is needed to initiate appropriate empirical treatment.

## Introduction

The placement of a drainage catheter in the surgical site is common in surgical procedures [1,2]. Although a surgical drainage catheter is expected to reduce postoperative collections and identify postoperative complications, it facilitates the entry of bacteria into the clean wound area [3,4]. Thus, surgical drainage catheters are associated with possible complications, such as surgical drain infections (SDIs) [5]. In addition, the longer the drain remains in the body, the more likely bacteria are to be isolated from the drainage fluid [6,7].

Although there are several studies on surgical site infections (SSIs) in the literature, few of these have focused on the causative pathogenic agents of SDIs [8-12]. To the best of our knowledge, there are limited studies on antibiotic resistance patterns of microorganisms isolated from drain cultures. Periodic determination of antibiotic resistance can help formulate treatment strategies in the postoperative follow-up period.

The aim of our study was to identify bacterial species (spp.) and antimicrobial susceptibility in drain cultures, to determine the resistance patterns of the isolates, and to provide data to facilitate empirical treatment approaches, both in our hospital and in the wider region.

## Materials and methods

This was a retrospective study based on a 12-year data set. Drain culture samples from the intensive care unit and inpatient services of the University of Health Sciences, Samsun Training, and Research Hospital between January 2008 and January 2020 were included in the study. After approval from the institutional ethics review committee, the data were obtained from automated microbiology systems and the hospital’s information management system. Data such as microorganisms grown in drain cultures, antibiotic resistance rates, and demographic characteristics of patients were obtained from the hospital automation system.

Before aspirating the drainage fluid, the suction drains were sterilized with 10% aqueous povidone-iodine solution and aspirated with an aseptic technique. The drain culture samples were cultivated on 5% sheep blood agar and eosin methylene blue agar and chocolate agar. After incubation for 18-24 hours at 37°C, microorganism growth was determined using conventional methods. VITEK-2 (BioMérieux, France) and BD Phoenix (Becton Dickinson, USA) were used for automated identification and antibiotic susceptibility testing. The results were evaluated according to the standards of the Clinical and Laboratory Standards Institute and those of the European Committee on Antimicrobial Susceptibility Testing.

The analysis of the results was performed using the Predictive Analytics SoftWare (PASW) Statistics 18.0 for Windows (Statistical Package for the Social Sciences, SPSS Inc., Chicago, IL). Data are given as the mean and standard deviation. A chi-square test was used to compare categorical data, and the results are presented as frequencies and percentages.

## Results

Three hundred forty-six isolates were detected in samples obtained from 279 patients within the last 12 years. A single microorganism grew in drain culture samples from 230 patients. Polymicrobial growth was detected in samples from 49 (18%) patients. The mean age of the patients from whom the samples were obtained was 62.82 ± 17.77 years, of which 46% were females, and 54% were males. Two different microorganisms were isolated in 34 patients, three in 12 patients, and four different microorganisms in three patients. A total of 346 microorganisms, or isolates, were examined for antibiotic resistance.

Microorganism growth was most detected in drain culture samples received from intensive care units, followed by general surgery, and oncology services. Of the 279 patients with growth in the drain culture, 130 were being followed up in the intensive care unit (37.6%), 115 in the surgery service (33.2%), and 30 in the oncology service (8.7%). According to the distribution by year, SDIs were detected most frequently in the first three-year period (2008-2011) and the last three-year period (2016-2019) (n = 143, 44.3% and n= 125, 36.1%, respectively).

In the drain cultures, throughout the 12-year period, the most frequent isolates were microorganisms belonging to the *Enterobacteriaceae family* (n=153, 44.2%), followed by *Staphylococcus spp.* (n= 69, 19.9%), and *Acinetobacter baumanni *(n=43, 12.4%). In the final four-year period, microorganisms belonging to the *Enterobacteriaceae family *remained the most frequent isolates, followed by *Enterococcus spp.* Despite its rare growth, Candida *spp.* (n= 2, 0.6%), Corynebacterium *spp*., Alcaligenes spp., Aeromonas *spp.*, Achromobacter *spp.,* and Myroides odotorium isolates were also identified. Among the *Enterobacteriaceae family*, *Escherichia coli *(n= 72, 48%), Klebsiella spp. (n= 36, 24%), and *Enterobacter spp.* (n= 26, 17.33%) were the most frequent isolates.

In the *Enterobacteriaceae family*, the highest resistance was detected against ampicillin (95%), ampicillin-sulbactam (87%), and first- (85%), second- (76%), and third-generation (67%) cephalosporins. Besides, in the *Enterobacteriaceae* family, the least resistance rate was detected against amikacin and tigecycline (5% and 7%, respectively). Extended-spectrum beta-lactamase resistance was detected in 75 (49%) *Enterobacteriaceae* family isolates and resistance to carbapenems was detected in 17% of these isolates. In the *A. baumannii* complex, the resistance spectrum was as follows: gentamicin, levofloxacin, imipenem, and meropenem at 84%, 82%, 80%, and 80%, respectively, with the least resistance to colistin and tigecycline (0% and 3% of isolates, respectively). No colistin resistance was found among *Acinetobacter spp.* and *Pseudomonas spp*. In relation to pseudomonas, the highest resistance was detected against cefepime and aztreonam and the least against colistin. Resistance to carbapenems was found in 76 (31.2%) gram-negative bacteria. The antibiotic resistance rates of the gram-negative microorganisms are given in Table 1.

**Table 1:** Antibiotic resistance rates of gram-negative microorganisms.

	Enterobacteriaceae family (n=153)		Acinetobacter spp. (n=43)		Pseudomonas spp. (n=35)	
Antibiotics	Resistance	Susceptible	Resistance	Susceptible	Resistance	Susceptible
	% n	% n	% n	% n	% n	% n
Aztreonam	57 25	43 19	-	-	70 19	30 8
Imipenem	16 15	84 78	80 31	20 8	26 8	74 23
Tobramycine	35 13	65 24	61 11	39 7	50 7	50 7
Netilmycine	30 8	70 19	71 5	29 2	50 4	50 4
Levofloxacin	39 32	61 50	82 28	18 6	39 9	61 14
Trimethoprim/sulfamethoxazole	44 61	56 77	63 26	37 15	-	-
Tigecycline	7 6	93 77	23 3	77 10	31 10	69 22
Colistin	-	-	0 0	100 17	0 0	100 8
Gentamicin	30 42	70 100	84 31	16 6	18 6	82 28
Amikacin	5 7	95 131	59 23	41 16	27 8	73 22
Meropenem	14 20	86 119	80 31	20 8	20 1	80 4
Ertapenem	17 22	83 110	57 4	43 3	46 13	54 15
Cefepim	56 75	44 58	-	-	71 12	29 5
Ceftriaxone	67 93	33 46	-	-	-	-
Ceftazidime	62 79	38 48	-	-	36 10	64 18
Cefoxitin	62 79	38 48	-	-	-	-
Cefuroxime	76 75	24 24	-	-	-	-
Cefazolin	85 99	15 18	-	-	-	-
Piperacillin/tazobactam	38 51	62 84	-	-	33 11	67 22
Amoxicillin/clavulanic	87 109	13 16	-	-	-	-
Ampicillin	95 114	5 6	-	-	-	-

The highest resistance among *Staphylococcus spp*. was detected against penicillin (82%), levofloxacin (57%), and erythromycin (56%) and the least against vancomycin (0%) and linezolid (2%). Methicillin resistance was found in 55% of *Staphylococcus aureus*. The highest resistance among *Enterococcus spp.* was against ciprofloxacin (46%) and ampicillin (45%). 11% of *Enterococcus spp.* isolates were resistant to vancomycin. The antibiotic resistance of gram-positive bacteria is shown in Table 2.

**Table 2:** Antibiotic resistance rates of gram-positive microorganisms.

	Staphylococcus spp. (n=69)		Enterococcus spp. (n=29)	
Antibiotics	Resistance	Susceptibility	Resistance	Susceptibility
	% n	% n	% n	% n
Levofloxacin	57 8	43 6	-	-
Trimethoprim/sulfamethoxazole	25 14	75 43	-	-
Ciprofloxacin	47 24	53 27	46 6	54 7
Gentamicin	39 22	61 34	-	-
Cefoxitin	55 37	45 30	-	-
Ampicillin	-	-	45 13	55 16
Clindamycin	32 19	68 41	-	-
Linezolid	2 1	98 63	4 1	96 25
Rifampicin	31 17	69 37	-	-
Teicoplanin	5 3	95 52	10 2	90 19
Erythromycin	56 36	44 28	-	-
Vancomycin	0 0	100 62	11 3	89 23
Penicillin	82 23	18 5	-	-

## Discussion

There are limited studies in the literature on the frequency of causative pathogens in SDIs, and those that have been published lack data on the antibiotic resistance profiles of these pathogens [8-12]. This study provides preliminary data that could fill the research gap in the literature on antibiotic surveillance in SDIs.

In previous research, polymicrobial growth was found in 40.6% of drain fluid cultures obtained after a pancreaticoduodenectomy [11]. In our study, the isolates were not specific to a particular operation. Although polymicrobial growth was not as high as that previously reported, the rate was still high (18%). In our study, in terms of polymicrobial growth, two different types of microorganisms were isolated in 34 patients, three types in 12 patients, and four types in three patients.

In surgical drain fluid, some previous studies [5,7,8,10,13-15] reported that gram-positive bacteria (coagulase-negative staphylococci, *S. aureus*, and *Enterococcus spp.*) were the most frequently isolated, whereas some others reported that gram-negative and gram-positive bacteria were equally distributed in surgical drain fluid [9,12]. Other studies suggested that gram-negative bacteria were detected most frequently [11,15]. In our study, gram-negative bacteria were most frequent (n = 243, 70.2%), with members of the *Enterobacteriaceae family* of gram-negative bacteria most common. Coagulase-negative staphylococci were the second most frequently isolated microorganisms, and the third most frequent pathogen was *A. baumanni*. However, in the last three-year period (2016-2019), the frequency of *Staphylococcus spp.* and *A. baumann*i decreased, whereas that of *Enterococcus spp*. increased. Previous studies on SDIs reported different pathogen frequency profiles. The frequencies detected in our study might be related to the implementation of different prophylactic antibiotic policies over time.

**Figure 1:**
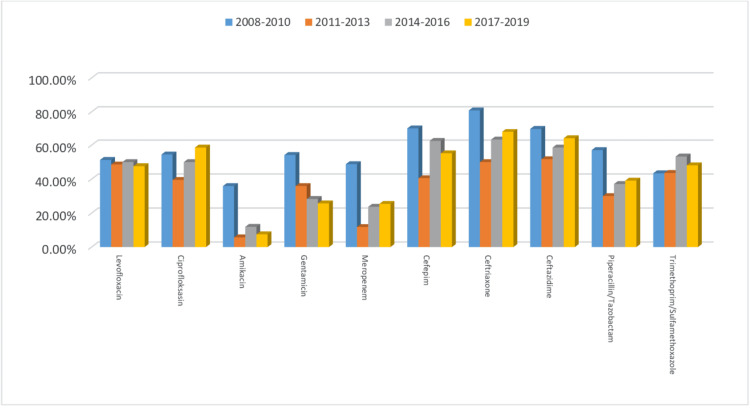
Antibiotic resistance rates in the different periods.

To better evaluate changes in antibiotic resistance rates over time, we divided the 12-year data set into three-year periods, starting from 2008. The highest antibiotic resistance rate was detected in the first three-year period, between 2008 and 2010. Although the resistance rates tended to decrease in the second three-year period, they increased again in subsequent periods. This decrease could be related to the number of isolates and antibiotic policies used in recent years. The antimicrobial resistance rates in the different periods are shown in Figure 1.

In surgical drainage fluids, methicillin resistance was found at a rate of 0%-68% in *S. aureus* strains isolated from drain cultures [5,8,16]. In our study, methicillin resistance was detected in 55% of *S. aureus*. The rate of methicillin resistance detected in our study is within the range found in other studies.

This study has some limitations. First, this study has a retrospective design and was conducted at a single center. Second, the effect of antibiotic resistance on clinical parameters and long-term follow-up was not fully evaluated.

## Conclusions

Antimicrobial susceptibility studies require considerable time. In SDIs, information on the antibiotic resistance rates of isolates from drain cultures can aid the selection of appropriate antibiotics and early initiation of treatment. Antibiotic use policies, especially empirical treatment, should be based on antimicrobial resistance surveillance. To obtain stronger evidence about the clinical value of drainage fluid cultures, further prospective clinical studies in a larger population focused on the type of surgery with detailed short and long-term clinical information should be performed.
